# Latent Tuberculosis and Associated Factors Among Adults Living With HIV in Kinshasa, Democratic Republic of the Congo: A Cross‐Sectional Study

**DOI:** 10.1002/puh2.70380

**Published:** 2026-09-15

**Authors:** José Mutambayi Kadima, Aliocha Natuhoyila Nkodila, Pascal Tshindele Lutumba, Jean‐René Kabangu M'buyamba, Placide Kingebeni Mbala, Jeremie Masidi Muwonga, Marie‐Joelle Sifa Muchanga, Daniel Mukadi‐Bamuleka, Marie‐José Bajani Kabedi, Mecky Isaac Matee, Jean Marie Ntumba Kayembe

**Affiliations:** ^1^ Department of Medical Biology University of Kinshasa Kinshasa Democratic Republic of the Congo; ^2^ Immunology and Microbiology Department Institute of Traditional Medicine Muhimbili University of Health and Allied Sciences Dar es Salaam United Republic of Tanzania; ^3^ Department of Family Medicine and Primary Health Care Protestant University in Congo Kinshasa Democratic Republic of the Congo; ^4^ Department of Tropical Medicine University of Kinshasa Kinshasa Democratic Republic of the Congo; ^5^ Department of Internal Medicine University of Kinshasa Kinshasa Democratic Republic of the Congo; ^6^ Department of International Trials Kokuritsu Kokusai Iryo Kenkyu Center Byoin Kokyuki Naika Shinjuku City Tokyo Japan; ^7^ Institut National de Recherche Biomédicale, INRB Kinshasa Democratic Republic of the Congo; ^8^ Rodolphe Mérieux INRB‐Goma Laboratory Goma Democratic Republic of the Congo

**Keywords:** associated factors, Democratic Republic of the Congo, human immunodeficiency virus (HIV), latent tuberculosis

## Abstract

**Background:**

Latent tuberculosis infection (LTBI) is more frequent among people living with human immunodeficiency virus (HIV) (PLHIV), in whom immunosuppression favors reactivation. However, data from sub‐Saharan Africa remain limited. This study aimed to determine the prevalence of LTBI and identify its associated factors among adults living with HIV in Kinshasa.

**Methods:**

An analytical cross‐sectional study was conducted between May 2022 and October 2025 in nine HIV care centers in Kinshasa. Adult HIV‐positive patients were consecutively recruited. LTBI was screened using the tuberculin skin test (TST) and/or interferon‐gamma release assays (IGRAs). Socio‐demographic, clinical, and immunological data, including CD4 cell counts, were collected. Associated factors were identified using multivariate logistic regression at a significance level of *p* < 0.05.

**Results:**

Among 248 participants, 74.7% were women. The mean age was 45.5 ± 10.7 years, and the median CD4 count was 321 cells/µL (IQR: 281–390). Overall, 25.8% were IGRA‐positive and 30.6% TST‐positive, with 38.3% indeterminate IGRA results. The main factors associated with LTBI were CD4 count <200 cells/µL (IGRA: adjusted odds ratio [aOR] = 3.34; TST: aOR = 3.64), history of positive TST (IGRA: aOR = 2.50; TST: aOR = 2.78), and contact with an active TB case (IGRA: aOR = 2.94).

**Conclusions:**

The prevalence of LTBI among adults living with HIV in Kinshasa is high. Severe immunosuppression, prior exposure, and contact with active TB cases are key determinants. These findings highlight the importance of screening and preventive therapy to reduce progression to active tuberculosis.

## Graphical abstract

Latent tuberculosis infection is common among adults living with HIV in Kinshasa. Advanced immunosuppression, previous TB exposure, and contact with active TB cases were independently associated with LTBI, supporting systematic screening and targeted tuberculosis preventive therapy.

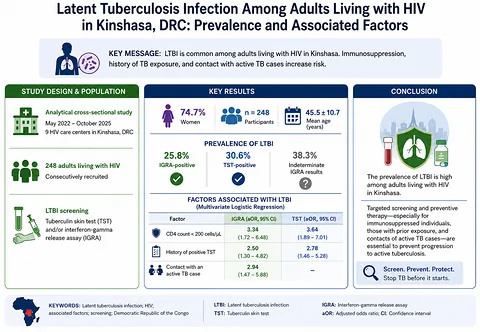

## 1 Introduction

Tuberculosis (TB) remains a major global public health concern and one of the leading causes of morbidity and mortality, particularly in low‐ and middle‐income countries [1]. It is estimated that nearly one‐quarter of the world's population is infected with *Mycobacterium tuberculosis* in the form of latent tuberculosis infection (LTBI), constituting a significant reservoir for future active TB cases [2]. LTBI is defined by an immune response to the bacillus without clinical or radiological evidence of active disease, but with a persistent risk of reactivation, especially among immunocompromised individuals [3]. Human immunodeficiency virus (HIV) infection is one of the strongest risk factors for progression from latent to active TB [4]. Among people living with HIV (PLHIV), progressive immune suppression, particularly CD4 T‐cell depletion, promotes reactivation of latent infection [1]. The annual risk of developing active TB among PLHIV with LTBI is estimated to range from 3% to 16%, significantly higher than in the general population [5].

In sub‐Saharan Africa, a region bearing a dual burden of TB and HIV, LTBI represents a major public health challenge [6]. Approximately 22% of the African population is estimated to carry LTBI, with higher prevalence observed among high‐risk groups such as PLHIV, healthcare workers, and populations living in densely populated urban settings [7, 8]. Several studies across African countries report LTBI prevalence among PLHIV ranging from 22% to over 50%, depending on diagnostic methods and population characteristics [1, 9]. Detection of LTBI primarily relies on the tuberculin skin test (TST) and interferon‐gamma release assays (IGRAs), which are essential for identifying individuals eligible for preventive treatment to reduce progression to active TB, particularly among PLHIV [10]. However, despite these strategies, systematic screening and management of LTBI remain insufficient in many high‐burden countries due to limited resources, restricted access to diagnostic tools, and health system constraints [11].

The Democratic Republic of the Congo (DRC) is among the countries with high TB incidence and HIV prevalence in Africa. In a context marked by high population density and unequal access to healthcare, PLHIV are at increased risk of both exposure to and reactivation of TB infection [12]. However, local data on LTBI prevalence and associated factors remain limited, underscoring the need for studies such as this one, which aims to estimate LTBI prevalence and identify associated factors among adults living with HIV in Kinshasa.

## 2 Methods

This analytical cross‐sectional study was conducted in Kinshasa from May 14, 2022 to October 16, 2025, in a context marked by high population density and a dual burden of HIV and TB. The study took place in HIV care facilities providing antiretroviral therapy and routine clinical follow‐up. From an initial list of 318 HIV care centers, 30 were randomly selected, and nine ultimately agreed to participate. These centers offered comprehensive HIV services, including clinical consultation and immunological monitoring. The study population consisted of adults (≥18 years) living with HIV who were followed in the selected centers and provided informed consent. Participants were consecutively recruited during routine visits from PLHIV attending the HIV outpatient clinic between May 14, 2022 and October 16, 2025. Consecutive sampling was used to reduce selection bias by enrolling all eligible patients presenting during the study period. Adults (≥18 years) with confirmed HIV infection who attended routine HIV care during the study period and provided written informed consent were eligible for inclusion. Participants were required to have undergone screening for LTBI using TST and/or IGRA according to routine clinical practice. Patients with confirmed or suspected active TB at enrollment, those receiving anti‐TB treatment or TB preventive therapy, pregnant women (if applicable), individuals unwilling or unable to provide informed consent, and those with incomplete key clinical or laboratory data and individuals with acute HIV infection were not included in the study.

Data were collected using a standardized questionnaire administered by trained healthcare personnel. Information included socio‐demographic characteristics (age, sex, marital status), clinical history (diabetes, prior TB, contact with TB cases, previous positive TST), and biological parameters, particularly CD4 cell count, and information on antiretroviral therapy (ART), including treatment status, duration of therapy, and ART regimen, was extracted from participants’ medical records and incorporated into the analysis. Screening for LTBI was performed using the TST and/or the IGRA, according to test availability and routine clinical practice during the study period. Both tests were considered because they provide complementary diagnostic information. Although TST is inexpensive and widely available, its sensitivity may be reduced in PLHIV due to impaired cell‐mediated immunity, and its specificity may be affected by prior Bacillus Calmette–Guérin (BCG) vaccination. In contrast, IGRA is more specific in BCG‐vaccinated populations because it is not influenced by previous vaccination, although its performance may also decline in individuals with severe immunosuppression and its higher cost limits universal implementation. In accordance with current WHO recommendations, either TST or IGRA can be used for LTBI screening depending on local resources and the epidemiological setting. Therefore, both tests were accepted in this study to reflect routine clinical practice and optimize LTBI detection among PLHIV.

IGRA results were interpreted according to the manufacturer's instructions. An indeterminate result was considered when either the negative control or positive control failed to meet the required validity criteria. Whenever feasible, participants with an indeterminate IGRA result underwent repeat testing using a newly collected blood sample. If repeat testing remained indeterminate or could not be performed, the result was classified as indeterminate and excluded from analyses requiring definitive classification of LTBI. The management of indeterminate results followed the manufacturer's recommendations and international guidelines for the diagnosis of LTBI in PLHIV.

### 2.1 Statistical Analysis

The collected data were entered and analyzed using SPSS for Windows version 25. Quantitative variables were described using the mean and standard deviation or the median and interquartile range, depending on data distribution, whereas qualitative variables were presented as frequencies and percentages. The prevalence of latent TB was estimated with its 95% confidence interval. The analysis of associated factors was first performed using bivariate analysis, applying the chi‐square test or Fisher's exact test for qualitative variables and Student's *t*‐test or the Mann–Whitney test for quantitative variables. Variables with a *p* value less than 0.05 in the bivariate analysis were included in a multivariate logistic regression model to identify factors independently associated with latent TB. Results were expressed as adjusted odds ratios (aOR) with their 95% confidence intervals, and the level of statistical significance was set at *p* < 0.05.

## 3 Results

Among the study participants, 100.0% were receiving antiretroviral therapy at the time of enrollment. The median duration of ART was 10.0 (IQR: 9.0–11.1) years, and the most frequently prescribed regimen was ABC + 3TC + DTG (13.1%), TDF + 3TC + DTG (80.4%), and AZT + 3TC + DTG (6.5%).

### 3.1 General Characteristics of the Study Population

Table 1 presents the socio‐demographic and clinical characteristics of the 237 participants, including 60 men (25.3%) and 177 women (74.7%). The mean age was 45.5 ± 10.7 years, with men significantly older than women (46.5 ± 12.1 vs. 42.2 ± 10.1 years; *p* = 0.002). Most participants were aged 30–49 years (61.6%). Marital status differed significantly by sex (*p* = 0.003): men were more often married (44.3%), whereas women were more frequently widowed (34.2%). Almost all participants had received BCG vaccination (99.6%). Additionally, 27.0% reported contact with an active TB case, with no significant sex difference. Immunologically, the median CD4 count was 321 cells/µL (IQR: 281–390) and was significantly higher in women than men (338.5 vs. 306.5 cells/µL; *p* = 0.027). About 33.2% of participants had CD4 counts <200 cells/µL (Table 1).

**TABLE 1:** Distribution of general characteristics of the study population by sex.

Variables	Overall (*n* = 237)	Male (*n* = 60)	Female (*n* = 177)	*p*
Age (years)	45.5 ± 10.7	46.5 ± 12.1	42.2 ± 10.1	0.002
18–29 years	17 (7.2)	8 (13.3)	9 (5.1)	
30–49 years	146 (61.6)	26 (43.3)	120 (67.8)	
≥50 years	74 (31.2)	26 (43.3)	48 (27.1)	
Marital status				**0** **.003**
Married	80 (32.3)	27 (44.3)	53 (28.3)	
Single	87 (35.1)	24 (39.3)	63 (33.7)	
Widowed	71 (28.6)	7 (11.5)	64 (34.2)	
Divorced	10 (4.0)	3 (4.9)	7 (3.7)	
History of positive TST				0.310
No	240 (96.8)	58 (95.1)	182 (97.3)	
Yes	8 (3.2)	3 (4.9)	5 (2.7)	
BCG vaccination				—
No	1 (0.4)	0 (0.0)	1 (0.5)	
Yes	247 (99.6)	61 (100.0)	186 (99.5)	
Contact with active TB case				0.377
No	181 (73.0)	46 (75.4)	135 (72.2)	
Yes	67 (27.0)	15 (24.6)	52 (27.8)	
HIV/AIDS	248 (100.0)	61 (100.0)	187 (100.0)	—
Diabetes mellitus (DM)	6 (2.4)	1 (1.6)	5 (2.7)	0.542
CD4 count (cells/µL)	321.0 (281.0–390.0)	306.5 (172.6–471.8)	338.5 (281.0–397.0)	**0.027**
<200	73 (33.2)	24 (42.9)	49 (29.9)	
≥200	147 (66.8)	32 (57.1)	115 (70.1)	

Abbreviations: BCG, Bacillus Calmette–Guérin; CD4, cluster of differentiation 4; HIV, human immunodeficiency virus; TB, tuberculosis; TST, tuberculin skin test.

### 3.2 Prevalence of Latent TB

Among the 248 participants, 25.8% (95% CI: 20.2–31.5) had a positive IGRA, whereas 35.9% (95% CI: 29.8–42.3) were negative and 38.3% (95% CI: 32.7–44.4) had indeterminate results. Regarding the TST, 30.6% (95% CI: 25.4–36.7) of participants had a positive result, compared to 69.4% (95% CI: 63.3–74.6) with negative results (Table 2).

**TABLE 2:** Results of latent tuberculosis screening tests.

Results	IGRA (*n* = 248)	% (95% CI)	TST (*n* = 248)	% (95% CI)
Negative	89	35.9 (29.8–42.3)	172	69.4 (63.3–74.6)
Positive	64	25.8 (20.2–31.5)	76	30.6 (25.4–36.7)
Indeterminate	95	38.3 (32.7–44.4)	—	—

Abbreviations: CI, confidence interval; IGRA, interferon‐gamma release assay; TST, tuberculin skin test.

### 3.3 General Characteristics of Patients According to IGRA Results

Patients with positive IGRA results showed factors significantly associated with latent TB. They were more frequently single or widowed (marital status, *p* = 0.031), had a history of positive TST (9.4%; *p* = 0.002), reported contact with an active TB case (39.1%; *p* = 0.042), and had diabetes mellitus (6.3%; *p* = 0.018). They were also more immunosuppressed, with a lower median CD4 count (197.5 cells/µL), and 50% of patients had CD4 counts <200 cells/µL (*p* < 0.001) (Table 3).

**TABLE 3:** General characteristics of patients according to interferon‐gamma release assay (IGRA) results.

Variables	Negative (*n* = 84)	Positive (*n* = 62)	Indeterminate (*n* = 91)	*p*
Age (years)	46.2 ± 10.8	45.9 ± 10.9	44.6 ± 10.4	0.140
18–29 years	6 (7.1)	4 (6.5)	7 (7.7)	
30–49 years	49 (58.3)	36 (58.1)	61 (67.0)	
≥50 years	29 (34.5)	22 (35.5)	23 (25.3)	
Sex				0.734
Male	20 (22.5)	15 (23.4)	26 (27.4)	
Female	69 (77.5)	49 (76.6)	69 (72.6)	
Marital status				**0.031**
Married	33 (37.1)	15 (23.4)	32 (33.7)	
Single	25 (28.1)	24 (37.5)	38 (40.0)	
Widowed	28 (31.5)	22 (34.4)	21 (22.1)	
Divorced	3 (3.4)	3 (4.7)	4 (4.2)	
History of positive TST	2 (2.2)	6 (9.4)	0 (0.0)	**0.002**
Contact with active TB case	23 (25.8)	25 (39.1)	20 (21.1)	**0.042**
Diabetes mellitus	1 (1.1)	4 (6.3)	1 (1.1)	**0.018**
CD4 count (cells/µL)	336.0 (287.0–517.0)	197.5 (131.0–332.2)	412.0 (297.8–694.0)	**<0.001**
<200	24 (29.6)	32 (50.0)	17 (22.7)	
≥200	57 (70.4)	32 (50.0)	58 (77.3)	

Abbreviations: CD4, cluster of differentiation 4; TB, tuberculosis; TST, tuberculin skin test.

### 3.4 General Characteristics According to TST Results

The results in Table 4 show that participants with a positive TST exhibited certain characteristics significantly associated with latent TB. These patients were more often widowed and had a history of a positive TST (*p* = 0.013). Immunologically, they had a significantly lower median CD4 count (191 cells/µL), and 53.8% of them had CD4 counts <200 cells/µL (*p* < 0.001) (Table 4).

**TABLE 4:** General characteristics according to tuberculin skin test (TST) results.

Variables	TST negative (*n* = 160)	TST positive (*n* = 52)	*p*
Age (years)	45.5 ± 11.4	45.4 ± 10.7	0.281
18–29 years	14 (8.8)	2 (3.8)	
30–49 years	91 (56.9)	36 (69.2)	
≥50 years	55 (34.4)	14 (26.9)	
Sex			0.137
Male	45 (28.1)	10 (19.2)	
Female	115 (71.9)	42 (80.8)	
Marital status			0.021
Married	52 (32.5)	13 (25.0)	
Single	62 (38.8)	18 (34.6)	
Widowed	41 (25.6)	18 (34.6)	
Divorced	5 (3.1)	3 (5.8)	
History of positive TST	4 (2.5)	4 (7.7)	0.013
Contact with active TB case	42 (26.3)	17 (32.7)	0.092
Diabetes mellitus	5 (3.1)	1 (1.9)	0.544
CD4 count (cells/µL)	379.0 (307.1–477.0)	191.0 (118.0–281.0)	<0.001
<200	42 (26.3)	28 (53.8)	
≥200	118 (73.8)	24 (46.2)	

*Note:* Values are expressed as mean ± standard deviation or median (interquartile range), and frequencies (percentages).

Abbreviations: CD4, cluster of differentiation 4; TB, tuberculosis.

### 3.5 Factors Associated With Latent TB

Multivariate analysis identified several factors significantly associated with latent TB among adults living with HIV, measured by both TST and IGRA. For the TST, patients with CD4 counts <200 cells/µL had a significantly higher risk of latent TB (aOR = 3.64; 95% CI: 1.89–7.02; *p* < 0.001). Additionally, a history of a positive TST was also associated with an increased risk (aOR = 2.78; 95% CI: 1.09–6.21; *p* = 0.040).

For IGRA, CD4 counts <200 cells/µL remained a major risk factor (aOR = 3.34; 95% CI: 1.76–6.33; *p* < 0.001). Patients who had contact with an active TB case also had a significantly higher risk (aOR = 2.94; 95% CI: 1.98–5.83; *p* = 0.016). A history of positive TST was additionally associated with a positive IGRA result (aOR = 2.50; 95% CI: 1.08–5.98; *p* = 0.041) (Table 5).

**TABLE 5:** Factors associated with latent tuberculosis.

Variables	TST				IGRA			
	Univariate analysis		Multivariate analysis		Univariate analysis		Multivariate analysis	
	*p*	OR (95%CI)	*p*	aOR (95%CI)	*p*	OR (95%CI)	*p*	aOR (95%CI)
Marital status								
Married						1		1
Not married	—	—	—	—	**0.048**	1.78 (1.29–3.42)	0.172	1.64 (0.81–3.32)
History of positive TST								
No		1		1		1		1
Yes	**0.024**	2.33 (1.56–9.59)	**0.040**	2.78 (1.09–6.21)	**0.007**	9.41 (4.85–17.92)	**0.041**	2.50 (1.08–5.98)
Contact with active TB case								
No		1		1		1		1
Yes	**0.046**	2.02 (1.56–4.86)	0.556	1.24 (0.60–2.56)	**0.016**	2.10 (1.15–3.86)	**0.016**	2.94 (1.98–5.83)
Diabetes mellitus								
No		1		1		1		1
Yes	**0.046**	0.45 (0.05–0.88)	0.297	0.23 (0.02–3.61)	**0.040**	2.07 (1.08–3.57)	0.341	2.75 (0.34–6.12)
CD4 count (cells/µL)								
≥200		1		1		1		1
<200	**<0.001**	3.41 (1.81–6.42)	**<0.001**	3.64 (1.89–7.02)	**0.001**	2.81 (1.53–5.14)	**<0.001**	3.34 (1.76–6.33)

Abbreviations: aOR, adjusted odds ratio; CD4, cluster of differentiation 4; CI, confidence interval; IGRA, interferon‐gamma release assay; TB, tuberculosis; TST, tuberculin skin test.

## 4 Discussion

This study conducted among 248 adults living with HIV in Kinshasa found that 25.8% of participants had a positive IGRA, whereas 30.6% had a positive TST. A notable finding was the high proportion (38.3%) of indeterminate IGRA results, reflecting the challenges of diagnosing LTBI in immunocompromised populations. The prevalence of LTBI identified by IGRA falls within the moderate range reported in sub‐Saharan Africa, where estimates among PLHIV generally vary from 20% to 60% depending on epidemiological context and healthcare access [13, 14]. The high rate of indeterminate IGRA results is consistent with previous studies, as severe immunosuppression can impair interferon‐gamma (IFN‐γ) production by T lymphocytes, leading to inconclusive results [15].

The slightly higher positivity rate observed with TST compared to IGRA may be explained by differences in their underlying mechanisms. Although IGRA measures IFN‐γ production by antigen‐specific T cells, TST relies on a delayed‐type hypersensitivity reaction. In individuals with moderate immunosuppression, TST responses may remain detectable even when IFN‐γ production is reduced, resulting in higher positivity rates [16]. Discordance between TST and IGRA is well documented in PLHIV, with concordance rates ranging from 50% to 80%, and a higher frequency of indeterminate IGRA results in patients with CD4 counts below 200 cells/mm^3^ [17, 18]. These findings highlight the complex interaction between HIV and *M. tuberculosis*. Approximately one‐third of participants had CD4 cell counts below 200 cells/µL, indicating advanced immunosuppression. This finding likely reflects delayed HIV diagnosis, advanced disease at presentation, incomplete immune recovery despite ART, or treatment interruption, all of which remain common challenges in many sub‐Saharan African settings. Progressive CD4 T‐cell depletion compromises immune responses, reducing the capacity to contain latent infection and increasing the risk of reactivation [19]. The observed prevalence underscores the need for systematic LTBI screening and the potential value of combining diagnostic tools or tailoring approaches based on immune status.

Multivariate analysis identified several factors independently associated with LTBI. Immune status, as reflected by CD4 cell count, was the most significant determinant. Patients with CD4 counts below 200 cells/µL had a markedly increased risk of LTBI, both by TST (aOR = 3.64; 95% CI: 1.89–7.02; *p* < 0.001) and IGRA (aOR = 3.34; 95% CI: 1.76–6.33; *p* < 0.001). This finding is consistent with the known pathophysiology of TB in HIV infection, where impaired cellular immunity facilitates both infection and reactivation [19]. Similar results have been reported in studies from Africa and Latin America, including Ethiopia and Brazil, where low CD4 counts were strongly associated with latent or active TB [20, 21, 22, 23].

A history of positive TST was also significantly associated with LTBI in this study, both for TST (aOR = 2.78; 95% CI: 1.09–6.21; *p* = 0.040) and IGRA (aOR = 2.50; 95% CI: 1.08–5.98; *p* = 0.041). This likely reflects persistent immunological memory following previous exposure to *M. tuberculosis*. Existing evidence supports that individuals with prior positive TST results are more likely to remain positive on subsequent tests, including IGRA, indicating ongoing latent infection [24, 25]. These findings emphasize the importance of incorporating patient history, particularly previous TB exposure or testing, into LTBI screening strategies. Contact with an active TB case was significantly associated with LTBI when assessed by IGRA (aOR = 2.94; 95% CI: 1.98–5.83; *p* = 0.016), but not by TST after adjustment. This suggests that IGRA, which is more specific to *M. tuberculosis* antigens and less influenced by BCG vaccination or environmental mycobacteria, may better capture recent or confirmed exposure [22, 23]. Similar findings have been reported in other settings, where documented TB contact increases the likelihood of LTBI detection, particularly by IGRA [26]. International guidelines also recognize prior exposure and positive TST history as key indicators for targeted screening and preventive therapy, especially among PLHIV [26].

Despite its contributions, this study has several limitations. Its cross‐sectional design precludes causal inference, limiting interpretation to associations. The limited availability of IGRA testing and reliance on TST in some settings may have influenced results, particularly the high rate of indeterminate IGRA outcomes. Some potentially important confounding factors, such as viral load, socioeconomic status, and nutritional status, were not fully assessed. Additionally, the lack of HIV viral load data prevented the assessment of virological suppression and its potential association with LTBI. CD4 cell count was therefore the only available marker of immune status. Future studies will incorporate viral load measurements to provide a more comprehensive evaluation of the relationship between HIV disease control and LTBI. Nevertheless, the study provides valuable insights into LTBI prevalence and associated factors among PLHIV in Kinshasa. These findings support the need for strengthened, context‐specific screening strategies and improved access to diagnostic tools to enhance TB prevention efforts in high‐burden settings.

## 5 Conclusion

The prevalence of latent TB among adults living with HIV in Kinshasa was high, with 25.8% IGRA‐positive and 30.6% TST‐positive results. Key associated factors included CD4 counts <200 cells/µL, a history of positive TST, and contact with an active TB case. These findings highlight the critical role of immunosuppression and prior exposure in latent infection detection and emphasize the need to strengthen preventive measures, including systematic screening and prophylaxis, to prevent progression to active TB in this high‐risk population.

## Author Contributions

José Mutambayi Kadima conceptualized the research topic. Aliocha Natuhoyila Nkodila and Pascal Tshindele Lutumba drafted the protocol with input from José Mutambayi Kadima for the methods, prepared the submission for institutional review board approval, supervised the data collection, and drafted the manuscript. Aliocha Natuhoyila Nkodila provided guidance for the statistical analysis. Jean‐René Kabangu M'buyamba, Placide Kingebeni Mbala, Jeremie Masidi Muwonga, Marie‐Joelle Sifa Muchanga , Daniel Mukadi‐Bamuleka, Marie‐José Bajani Kabedi, Mecky Isaac Matee, and Jean Marie Ntumba Kayembe provided content oversight for the manuscript. All authors read and approved the final manuscript.

## Funding

The authors have nothing to report.

## Ethics Statement

The study protocol was approved and validated by the Ethics Committee of the School of Public Health of the University of Kinshasa under no. ESP/CE/057/2015 and complies with the principles of the Declaration of Helsinki. The risks incurred by the participants in this study were supposed to be minimal, given that the vaccines used had already undergone clinical trials and been approved by scientists.

## Consent

Written informed consent was obtained from all the participants and/or their legally acceptable representatives. Non‐literate participants were accompanied by a literate peer of their choice. Participants under 18 years of age were accompanied by their parent or guardian. Their informed assents and consent from parent or guardian were requested and signed before the enrollment to the study. Participants had the right to provide consent or not and to withdraw from the study at any time during the interview, without having to provide a reason.

## Conflicts of Interest

The authors declare no conflicts of interest.

## Data Availability

The datasets during the current study are available from the corresponding author upon reasonable request.
